# Considering the ethics of big data research: A case of Twitter and ISIS/ISIL

**DOI:** 10.1371/journal.pone.0187155

**Published:** 2017-12-01

**Authors:** Elizabeth Buchanan

**Affiliations:** Center for Applied Ethics, Office of Research and Sponsored Programs, University of Wisconsin-Stout, Menomonie, Wisconsin, United States of America; Universitat Rovira i Virgili, SPAIN

## Abstract

This is a formal commentary, responding to Matthew Curran Benigni, Kenneth Joseph, and Kathleen Carley’s contribution, “Online extremism and the communities that sustain it: Detecting the ISIS supporting community on Twitter”. This brief review reflects on the ethics of big data research methodologies, and how novel methods complicate long-standing principles of research ethics. Specifically, the concept of the “data subject” as a corollary, or replacement, of “human subject” is considered.

## Introduction

Matthew Curran Benigni, Kenneth Joseph, and Kathleen Carley’s paper “Online extremism and the communities that sustain it: Detecting the ISIS supporting community on Twitter”[1] presents an Iterative Vertex Clustering and Classification (IVCC) model to identify ISIS/ISIL supporters among Twitter users. This method enables greater detection of specific individuals and groups in large data sets, with its enhanced capabilities to identify and represent following, mention, and hashtag ties. With the exponential spread of and access to social media across the globe, its uses to identify specific individuals within networks or organizations should not be a surprise. Nor should it be a surprise that police, law enforcement, and intelligence agencies are striving to become technologically savvy and sophisticated with social media and big data in their quest to identify and disrupt communications for law and security purposes. Samson [2] notes that these agencies are “rapidly coming to terms with the potency of social media and Internet-based communication, not solely as an extension of their own mass communication…but as a phenomenological source of intelligence.” Further, the VOX-Pol project [3], based in Dublin, Ireland, has been hosting training academies for law enforcement in focusing on the “role of the Internet in contemporary violent political extremism(s), including the online strategies of violent jihadis and the extreme right; the role of the Internet in lone actor terrorism; the online behaviours of convicted terrorists; and online CVE (Countering Violent Extremism).” Too, in the United States, we are seeing law enforcement and in particular, the FBI, as participants and co-sponsors to such events as the 3I conference, which focuses on research regulation, biosafety/biosecurity, and social media/big data [4], but other literature suggests that US efforts in various WebOps have been ineffective [5, 6].

Ethicists and privacy advocates, among others, have pushed back against large-scale data mining and analytics in the name of national intelligence and security but data have become so readily available—provided by the users themselves—the battle to protect individual liberties seems increasingly more challenging. Certainly, the Snowden revelations were just one of many recent incidents which reminded us of the fragility of privacy in an age of pervasive computing; too, recent events only exaggerate the growing role of online propaganda and sophisticated techniques employed by both state and non-state actors in online operations (and here, I thank Matthew, Kenneth, and Kathleen for their keen comments as this response was developed).

Here, Benigni et al remind us why this fight remains so critical for the privacy landscape and for an ongoing discourse around the ethics of data science, analytics, and big data. Specifically, Benigni et al’s work examines ISIS/ISIL, a twenty-first century terrorist organization, and the ways it has used twenty-first century Internet technologies, in nuanced ways, to recruit, promote, and increase participation across their followers [7]. While they embody a “retrograde religious philosophy [8], ISIS/ISIL’s methods and tactics exploit the networks of social media and ubiquitous computing of today’s Internet. They also take advantage of the fundamental nature of social media, that of promotion, sharing ideas and seeking an ever-larger network of followers.

Benigni et al describe their use of IVCC as a form of social network analysis within the context of terrorist activities. With over 100 million daily Twitter users, the 119,156 accounts analyzed in Benigni, et al, is indeed a small percentage. But, as ISIS/ISIL has occupied a significant space in the current landscape of global terrorism, the methods used here to systematically seek and identify supporters and sympathizers provide intelligence agencies, researchers, and others ample opportunity to explore these networks of agents and supporters. The research problem, as stated, is to “identify the set of users within the 119, 156 accounts that support ISIS in varying degrees,” and to demonstrate “new opportunities for intelligence and strategic communications experts to gain needed understanding in large populations susceptible to extremism.”

The context and foci of the research are well-defined. The methods are technically valid and reliable. The ethics of the methods, however, are less clear, and part of this is the novelty of this form of research. Ethics and methods are interdependent, and the rise of mass data mining across social media and the Internet has presented ethical dilemmas surrounding privacy, rights and autonomy, and such social justice issues as discrimination. To think about the ethics of IVCC and other forms of social network and big data analyses, we can ask to what end this methodology will be used, and who will use it? Who has access to data and the means to manipulate it? And, regarding context, if ISIS/ISIL was replaced as the object of study with Black Lives Matter sympathizers, what changes? Big data methodologies are not discriminate (though they can be, and currently are, used in discriminating ways) and algorithmic processing can be used to identify ISIS supporters as readily as they can identify WalMart shoppers or political dissidents. The methods can transcend context.

Certainly, these questions concerning the ethics of data mining methodologies are not new, and Benigni et al recognize these ethical limitations of big data and social data analyses, while recognizing the practical implications of their work. And, rightfully, they encourage more ongoing policy debate surrounding big data mining, its uses, and consequences. And, a pointed and important ethical consideration must be: What are the implications and impacts of not conducting and reporting on these data?

We in the United States, as in the EU, are currently seeing more intentional analyses and ethical reflection around the ethics of big data, or real world data, with recent conferences and publications coming from the National Association of Education [9], the National Science Foundation and Computing Research Association [10], the Secretary’s Advisory Committee to the Office for Human Research Protections [11], among numerous others. As the EU General Data Protection Regulation goes into effect in 2018, researchers will be among those challenged by big data analyses and analytics and their uses in relation to individual and societal privacy.

Big data analyses can be retrospective, prospective (predictive), synchronous or asynchronous, relational and/or causal; big data science is meant to identify patterns, structures, and/or anomalies in large data sets. Big data science is now employed in virtually all disciplines, from education to epidemiology to criminal justice. With Twitter’s massive numbers of active accounts and active users, combined with its openness for researchers to explore and exploit its data, we can presume that research with big data in general, with Twitter being just one of many sources, will continue to grow and push boundaries on traditional research methods and ethics principles. Indeed, social network data and big data research fit awkwardly within western models of research ethics, which prioritize the individual, and affords the individual autonomy through informed consent. Further, individuals in research must be treated in an ethical manner by respecting their decisions and protecting them from harm, and ultimately, researchers are expected to secure and protect their participants’ well-being. From a US-regulatory perspective, researchers would conclude that seeking informed consent from all 119, 156 participants is “impracticable” and studies of this sort involve no more than minimal risk to those involved; some would assert that the data were publicly available and thus, exempt from further ethical review and regulation. It is, however, challenging to predict and plan for the “downstream harms” associated with IVCC and related methodologies.

Benigni et al’s paper operates on the condition that these data are accessible to researchers, law enforcement, and others; the accounts from which data are mined are public (open) accounts, and ultimately, identifying those vulnerable to, or susceptible to online extremism is itself a social benefit, a laudable goal. Indeed, the recruitment and encouragement of terrorist sympathizers is a public policy concern, and perhaps this method of analyses is a way forward in curbing such trends across social media platforms such as Twitter. And, here is where, from an ethical perspective, this paper pushes us to consider the complex relationships between and among research questions, methods, and uses of research data. When I first read an earlier version of this paper, I asked for ethics to be “frontloaded” into the discussion. I don’t think I was clear on what that might look like. I questioned the objectives of the paper: is this paper about Twitter communities of ISIS/ISIL supporters and sympathizers? Is it about a methodology that could be employed in any setting, with any population of concern? Is it, or could it, be about ethical presumptions in big data science?

To this last point, Benigni et al differentiate between “marketing [and] intelligence objectives” in big data research. The intent of the analyses matters, as they note the reasonable person standard with regards to privacy (“reasonable expectations of privacy”) in conjunction with the intent of the research. Thus, one may implicitly agree to one’s data sources being used for marketing purposes while that same person would not want their data used in intelligence gathering. But, big data research does not necessarily provide us with the opportunity to consent to either use, regardless of the intent. Big data research operationalizes large data sets, but can reveal much information about an individual and his/her networks of relations. It is cliché to comment at this point in time about surveillance states and societies, as we’ve actively contributed to such conditions, with never ceasing data streams and acquiescence to pervasive data collection in the name of convenience or efficiency. We’ve become data points, data subjects, as beautifully illustrated in Benigni’s graphics and mappings.

It is not surprising, that in proposed changes to the United States Common Rule [12], the federal regulations governing human research protections, advances to the research infrastructure were identified as a major driver in the revisions:

Since the Common Rule was promulgated, the volume and landscape of research involving human subjects have changed considerably. Research with human subjects has grown in scale and become more diverse. Examples of developments include: an expansion in the number and types of clinical trials, as well as observational studies and cohort studies; a diversification of the types of social and behavioral research being used in human subjects research; increased use of sophisticated analytic techniques to study human biospecimens; and the growing use of electronic health data and other digital records to enable very large datasets to be rapidly analyzed and combined in novel ways. Yet these developments have not been accompanied by major change in the human subjects research oversight system, which has remained largely unaltered over the past two decades.

The last time the United States federal regulations around human research protections were revised was 1991, and were not reflective of the emerging technological changes just beginning to affect the research enterprise.

However, the changes to federal regulations now clearly acknowledge the impact of technological changes:

Evolving technologies—including imaging, mobile technologies, and the growth in computing power—have changed the scale and nature of information collected in many disciplines. Computer scientists, engineers, and social scientists are developing techniques to integrate different types of data so they can be combined, mined, analyzed, and shared. The advent of sophisticated computer software programs, the Internet, and mobile technology has created new areas of research activity, particularly within the social and behavioral sciences.

The shifting research landscape is complex; data come from a myriad of sources, some intentional and some unintentional. We see more research bystanders, or collateral subjects, in these complex streams of data. One’s connections in a social media landscape matter, even those distant and impersonal. Human subjects research, as broadly understood, is fundamentally different in the age of data science. Methods such as IVCC rely on continuous data streams and analytics. Many of these data mining and analytic studies are considered “secondary analysis.” The degree to which a researcher has access to identifiable data, or the ability to ascertain information about the individual through, for example, reidentification techniques, are used as determinants of the level of risk and benefit in the current US regulatory model of the Common Rule.

And, Benigni et al acknowledge the shift towards an individual data subject: “…as opposed to using information about individuals to build networks, we now use networks to gain insight into individuals.” In this paper, are there 119,156 individual subjects, or are they a group, a collective data subject? Is an individual the sum of their collective accounts, scattered across social media, ready to be processed by ever smarter algorithms? A common concern I’ve had in light of Internet research, social network analyses, and now big data research involves the displacement of both researchers and their participants, or subjects, from each other and from contextualized meaning; the latter is becoming further distant from those studying them, which in odd ways, is an ethical step back from the methods of, for example, community-based participatory research, where the role of the participant is primary and central. We can be subjects, in marketing research, or intelligence activities, or behavioral interventions, and never know. Do data subjects have the right to consent and how would they? Are data subjects afforded the same rights and responsibilities as human subjects have been in the discourses of research ethics?

Notably, PLOSONE is extremely diligent in its concerns and enforcement of research ethics principles, with mandatory disclosures of ethics board review, for example. Begnini et al had no human subjects disclosures, which is common for big data research in general and with Twitter data in particular. Ethics boards are engaging with difficult questions surrounding big data. Benigni et al provide a suitable case for us to think about this changed, and ever changing, research landscape.

Why, as a reviewer, did I want Benigni et al’s paper published, despite my own philosophical reservations to the uses of big data analytics across social networks for ideological/political purposes? Benigni et al convinced me of the validity of their methods and of the current relevance of their work. They showed how data and data subjects can be understood, and misunderstood. They addressed the ethical challenges of this research, and called attention to dual-use, or multi-use data, an area of only increasing importance. They challenged extant models of human subjects research, and as the research environment continues to rely on multiple channels and creators of data, of information, they reminded us of the many actors, state and non-state, and ethical dilemmas in this new terrain. Indeed, tensions surround the very definition of state [13], further complicating this research.

The complexities of this seemingly unfettered research domain, these research methods and ethics, and the increasingly diluted spaces of social media and big data are daunting. The philosophical and practical questions are countless. I am left with a few, maybe simple questions. How do data scientists, such as Benigni et al, consider those 119,156 Twitter users? I ask again, are they subjects, participants? Is this really a new category, the data subject, and do data subjects have rights? Who has the authority and power to decide how and in what contexts IVCC and related mining methods are used, and to what end, in what context? As data science flourishes, let’s be sure to frontload the ethics of research.
